# Patterns of Neural Functional Connectivity in Infants at Familial Risk of Developmental Dyslexia

**DOI:** 10.1001/jamanetworkopen.2022.36102

**Published:** 2022-10-27

**Authors:** Xi Yu, Silvina Ferradal, Jade Dunstan, Clarisa Carruthers, Joseph Sanfilippo, Jennifer Zuk, Lilla Zöllei, Borjan Gagoski, Yangming Ou, P. Ellen Grant, Nadine Gaab

**Affiliations:** 1 State Key Laboratory of Cognitive Neuroscience and Learning, Beijing Normal University, Beijing, China; 2 Fetal Neonatal Neuroimaging and Developmental Science Center, Boston Children’s Hospital, Boston, Massachusetts; 3 Department of Intelligent Systems Engineering, Indiana University, Bloomington; 4 Laboratories of Cognitive Neuroscience, Division of Developmental Medicine, Department of Medicine, Boston Children’s Hospital, Boston, Massachusetts; 5 Department of Speech, Language & Hearing Sciences, Boston University, Boston, Massachusetts; 6 A.A. Martinos Center for Biomedical Imaging, Massachusetts General Hospital, Boston; 7 Department of Radiology, Boston Children’s Hospital, Boston, Massachusetts; 8 Harvard Medical School, Boston, Massachusetts; 9 Harvard Graduate School of Education, Cambridge, Massachusetts

## Abstract

**Importance:**

Developmental dyslexia is a heritable learning disability affecting 7% to 10% of the general population and can have detrimental impacts on mental health and vocational potential. Individuals with dyslexia show altered functional organization of the language and reading neural networks; however, it remains unknown how early in life these neural network alterations might emerge.

**Objective:**

To determine whether the early emergence of large-scale neural functional connectivity (FC) underlying long-term language and reading development is altered in infants with a familial history of dyslexia (FHD).

**Design, Setting, and Participants:**

This cohort study included infants recruited at Boston Children’s Hospital between May 2011 and February 2019. Participants underwent structural and resting-state functional magnetic resonance imaging in the Department of Radiology at Boston Children’s Hospital. Infants with FHD were matched with infants without FHD based on age and sex. Data were analyzed from April 2019 to June 2021.

**Exposures:**

FHD was defined as having at least 1 first-degree relative with a dyslexia diagnosis or documented reading difficulties.

**Main Outcomes and Measures:**

Whole-brain FC patterns associated with 20 predefined cerebral regions important for long-term language and reading development were computed for each infant. Multivariate pattern analyses were applied to identify specific FC patterns that differentiated between infants with vs without FHD. For classification performance estimates, 99% CIs were calculated as the classification accuracy minus chance level.

**Results:**

A total of 98 infants (mean [SD] age, 8.5 [2.3] months; 51 [52.0%] girls) were analyzed, including 35 infants with FHD and 63 infants without FHD. Multivariate pattern analyses identified distinct FC patterns between infants with vs without FHD in the left fusiform gyrus (classification accuracy, 0.55 [99% CI, 0.046-0.062]; corrected *P* < .001; Cohen *d* = 0.76). Connections linking left fusiform gyrus to regions in the frontal and parietal language and attention networks were among the paths with the highest contributions to the classification performance.

**Conclusions and Relevance:**

These findings suggest that on the group level, FHD was associated with an early onset of atypical FC of regions important for subsequent word form recognition during reading acquisition. Longitudinal studies linking the atypical functional network and school-age reading abilities will be essential to further elucidate the ontogenetic mechanisms underlying the development of dyslexia.

## Key Points

**Question:**

Are functional topologies of language- and reading-related cerebral regions in infancy associated with a familial history of developmental dyslexia?

**Findings:**

In a cohort study examining the resting-state functional connectivity in 98 infants during natural sleep, distinct functional connectivity patterns of the left fusiform gyrus were observed in infants with vs without familial risk of dyslexia. These differences were evident despite comparable behavioral, socioeconomic, and environmental characteristics between the groups.

**Meaning:**

These findings suggest that familial history of dyslexia was associated with alterations in infant functional connectivity of key regions important for subsequent word recognition, suggesting a potential early emergence of an atypical functional network underlying reading development.

## Teaser

This cohort study examines functional connectivity in language- and reading-related regions of the brain in infants with vs without familial history of developmental dyslexia.

## Introduction

Learning to read requires linking oral and written language,^1^ and therefore relies critically on the establishment of efficient communication among the language network, comprised of left frontal and temporoparietal regions of the brain, and the occipitotemporal visual pathways.^2^ Despite having typical hearing and vision, approximately 7% to 10% of children fail to develop typical word reading and decoding abilities, termed *developmental dyslexia*.^3^ The prevalence is higher, ranging from 40% to 60%, among children with familial history of dyslexia (FHD) than among those without FHD,^4^ suggesting a genetic basis.

Multiple dyslexia susceptibility genes have been identified^5^ and most have been hypothesized to impact early brain development,^6,7^ potentially through atypical formation of cortical connectivity patterns underlying the foundations of reading acquisition.^8,9^ However, the specific neural mechanisms require further investigation.^10^ Children with dyslexia are typically diagnosed after prolonged learning struggles^11^ and often exhibit persistent academic and psychosocial difficulties that can negatively impact their mental health and vocational potential.^12^ Importantly, children at risk for dyslexia can be identified before reading onset^13^ and interventions are more effective in young children.^14,15^ Therefore, it is critical to unravel the early developmental mechanisms of dyslexia, which are essential for developing early identification and intervention strategies for children at risk.

Neuroimaging studies have suggested atypical neurobiological mechanisms in large-scale functional organization underlying the development of dyslexia. Individuals with dyslexia not only show neural deficits in regions important for language and reading,^16,17,18^ but also exhibit disrupted connectivity among these regions and their connections with domain-general cortices.^19,20,21,22,23^ Importantly, both regional and connectivity differences are evident in prereaders who subsequently develop dyslexia,^24,25,26^ indicating that at least some of these neural alterations predate reading onset and do not result from reading failure.

Prospective longitudinal studies in children with FHD provide a unique opportunity to examine the developmental trajectories of the neurobiology of dyslexia in relation to a familial risk. Atypical neural characteristics in language and visual processing areas are reported in prereaders with FHD,^27,28,29,30^ as early as in infancy.^11,31,32^ While not all children with FHD develop dyslexia, as a group, they show poorer reading skills than children without FHD.^4^ Moreover, neural alterations observed in children with FHD are associated with subsequent reading skills,^31,32,33,34^ suggesting the early onset of atypical brain mechanisms underlying familial risk of dyslexia. However, previous studies primarily focused on the early deficits in local (regional) brain characteristics. Given the fundamental role of functional network mechanisms in typical and atypical reading acquisition, it is important to examine whether the early development of large-scale functional connectivity might be altered as a function of familial risk of dyslexia.

Using resting-state functional connectivity (FC) techniques, this study examined the intrinsic neural functional organization of the infant brain among 98 infants with and without FHD during natural sleep. We computed whole-brain FC patterns associated with each region important for language and reading development, given the strong associations between a region’s FC and its functional specialization over development.^35,36^ Using this approach, we have previously demonstrated prospective associations between infant FC and school-age language and foundational literacy abilities,^37^ suggesting an early neural scaffold for subsequent literacy development. Multivariate pattern analyses were applied to identify specific FC patterns that may differentiate between infants with vs without FHD. We hypothesized that regions playing important roles in subsequent language and literacy acquisition would show atypical FC patterns in infants with FHD compared with those without.

## Methods

This cohort study was approved by the institutional review boards at Boston Children’s Hospital and Harvard University. Before participation, informed written consent was obtained from a parent or legal guardian of each infant. The study followed the Strengthening the Reporting of Observational Studies in Epidemiology (STROBE) reporting guideline for cohort studies.

### Participants

Infants aged 4 to 13 months were included in analyses (eAppendix in the Supplement). Infants with at least 1 first-degree relative with a dyslexia diagnosis or reading difficulties (RD), as determined by parent questionnaires, were classified as having FHD. The infants without FHD were classified as controls.

### Behavioral and Environmental Characterization

Infants’ developmental milestones in gross motor, fine motor, visual reception, and receptive and expressive language domains were assessed using the Mullen Scales of Early Learning assessment.^38^ Parents of all participants completed questionnaires documenting the home language and literacy environment (HLE)^39^ and socioeconomic status, in terms of parental education (eTable 1 in the Supplement).

### Imaging Acquisition and Preprocessing

We adopted the same acquisition and preprocessing protocols as applied in a 2021 study by Yu et al^37^ and described in the eAppendix in the Supplement. In brief, a T1-weighted image and resting-state images were acquired from every infant and preprocessed using an infant-appropriate pipeline for subsequent group-level classification analyses.

### Classification Analyses of the FC Patterns Associated With Long-term Language and Reading Development

**Figure 1.**
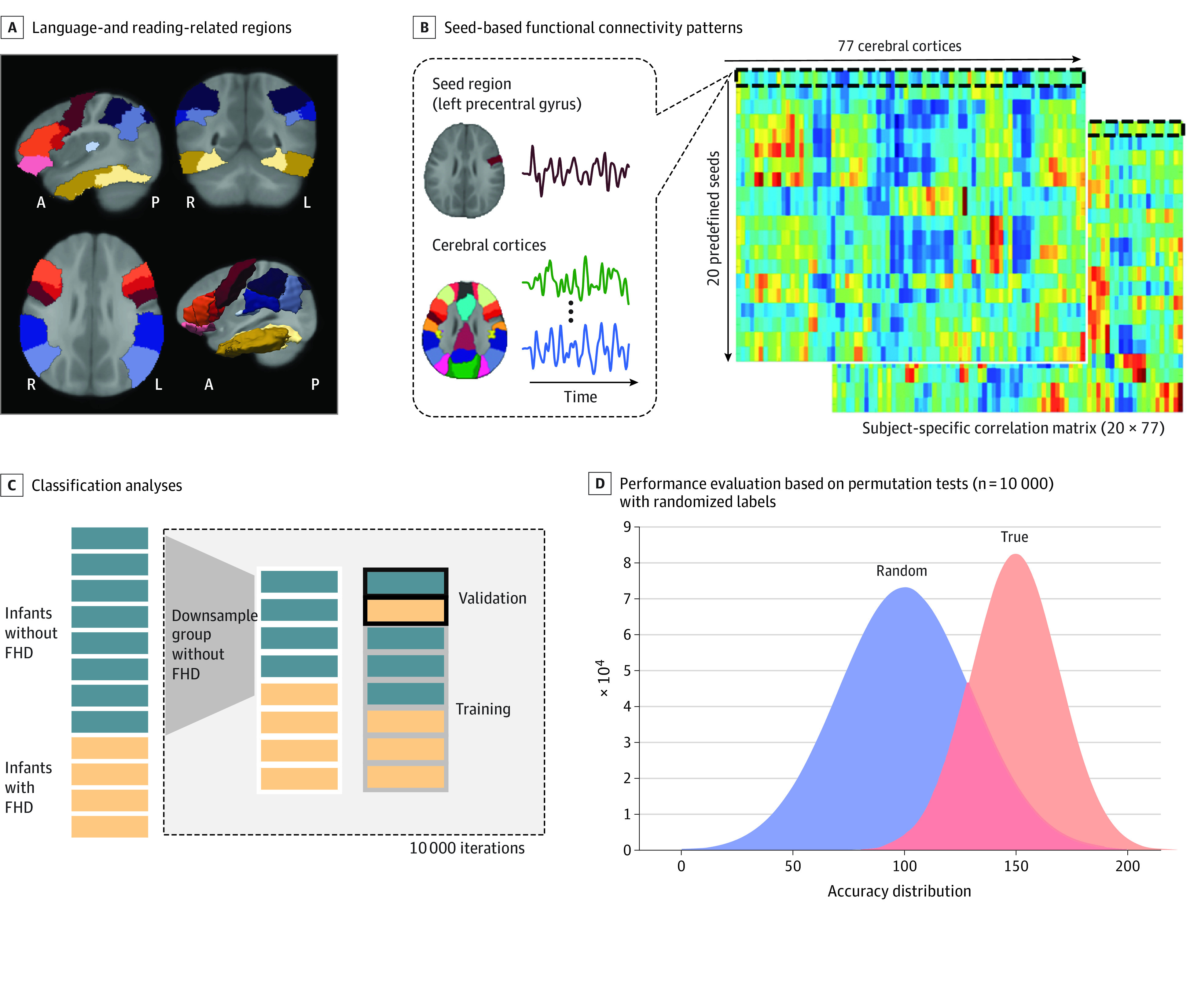
Flowchart Showing the Classification Analyses for Identifying Distinct Functional Connectivity (FC) Patterns Between Infants With vs Without Family History of Dyslexia (FHD) A, Slice views and 3D projections of 20 seed regions that were previously identified to be important for language and reading development. These include the inferior frontal (orbitofrontal, triangular, and opercular), precentral, temporoparietal (Heschl, inferior parietal, supramarginal, and angular) and occipitotemporal (inferior temporal and fusiform) cortices. Brain orientations are marked at the bottom of each image. A indicates anterior; L, left; P, posterior; and R, right). B, Computation process of the seed-based FC patterns for each infant. Specifically, region-specific time series were first estimated by calculating the mean of the BOLD signal across all the voxels in each of the cerebral regions defined based on the One-Year-Old Infant Brain Atlases^42^ derived from the Automated Anatomical Labeling atlas.^45^ Seed-specific FC patterns were then generated by correlating the time series of each seed region (eg, left precentral gyrus) with each of the other cerebral regions, and the correlation coefficients were normalized to *z*-scores using Fisher transformation. C, Classification analyses applied to identify regions with distinct FC patterns between infants with vs without FHD. Note that a bootstrapping approach was adopted to generate a balanced sample of 35 infants with FHD and 35 infants without FHD (randomly selected from 63 infants without FHD) for the classification analyses, which repeated 10 000 times to reduce sampling bias. D, Evaluation of classification results against a null distribution derived from the permutation tests where the same classification analyses were performed but with randomized labels. Result significance levels were Monte-Carlo corrected for multiple comparisons (n = 20).

Given our study’s focus on the early neural mechanisms underlying reading development, seed regions were placed in the frontal and temporoparietal language network and the occipitotemporal visual pathways,^40,41^ defined based on One-Year-Old Infant Brain Atlases (Figure 1A).^42^ Both hemispheres were considered, given their joint involvement during reading activities in individuals at risk for^43^ and diagnosed with dyslexia,^16^ as well as early reading development.^44^ Detailed classification analysis procedures are presented in the flowchart in Figure 1. Seed-specific FC patterns were generated by computing the Fisher-transformed correlations on the blood oxygenation level dependent timeseries between this region and each of the remaining cerebral regions (Figure 1B).

Classification analyses were performed using a support vector machine (SVM) classifier based on the LIBSVM package^46^ optimized using a linear kernel with default settings. The FC patterns of 1 seed were entered into the SVM analyses each time (Figure 1C). A leave-1-pair-out cross-validation strategy was used for estimating the accuracy of the classifier. A bootstrapping approach was adopted to achieve matched samples of the 2 groups, where 35 samples were randomly selected from the pool of infants without FHD to combine with the data of 35 infants with FHD. This process repeated 10 000 times to reduce sampling bias and generate a distribution of classification accuracies (ie, the true distribution). To evaluate the classification performance, a permutation test was conducted within each iteration, following the same procedure except that the FHD labels were randomly assigned, generating a null distribution over 10 000 iterations (Figure 1D). A paired *t* test was performed to evaluate whether the true distribution was significantly higher than the null distribution. Moreover, a distribution of the classification performance was computed for each seed by subtracting the classification accuracies of the permutation tests from those derived from the true labels within the same iteration. A 99% CI for the classification performance estimate was calculated to determine whether the lower CI bound was above 0, indicating better-than-chance-level classification performance, a similar approach to that applied in a study by Parkes et al.^47^ Finally, for the seed-specific FC patterns with significant *t* test results (corrected *P* < .05, Monte Carlo–corrected for multiple comparisons [n = 20]) and demonstrating a positive CI, the effect size was measured using Cohen *d*, in which larger magnitudes indicate stronger classification performance. Only seed regions showing a medium or large effect (*d* > 0.5)^48^ were considered as exhibiting distinct FC patterns between infants with vs without FHD.^48^ The classification sensitivity and specificity properties were also computed. Finally, for each region identified, we estimated the contribution of every feature (ie, FC) to the classification performance. Specifically, path-specific weights were first calculated from the bootstrapped samples (35 infants with FHD and 35 infants without FHD) used in each iteration, which were transformed into activation patterns of the corresponding forward model, using the equation described in Haufe et al.^49^ Mean pattern values across the 10 000 iterations were then generated for each path.

*P* values were 2-sided, and statistical significance was set at *P* < .05. Analyses were conducted using MATLAB version R2017b (MathWorks). Data were analyzed from April 2019 to June 2021.

## Results

The final data set included 98 infants (mean [SD] age, 8.5 [2.3] months; 51 [52.0%] girls), with 35 infants having FHD and 63 infants without FHD. Most infants with FHD had at least 1 parent with a dyslexia diagnosis or RD, except 4 infants who only had siblings with a dyslexia diagnosis (1 infant) or RD (3 infants). The 2 groups were balanced on age and sex (Table). No children had birth complications, neurological trauma, or known developmental delays. All infants’ structural images were interpreted as within expected clinical parameters by a pediatric neuroradiologist (P.E.G.).

**Table.** Demographic Characteristics and Head Movement During Scanning of Infants With and Without FHD

Measure	Mean (SD)		Group comparison	
	With FHD (n = 35)	Without FHD (n = 63)	*t*_96_	*P* value
Sex, No.				
Girls	15	36	1.84^a^	.17
Boys	20	27		
Age, mo	8.9 (2.4)	8.3 (2.3)	1.15	.25
Head movement during imaging acquisition				
FD, mm	0.083 (0.027)	0.082 (0.024)	0.22	.83
Outlier scans, %	7.6 (9.1)	6.7 (8.8)	0.49	.62

Abbreviations: FD, framewise displacement; FHD, familial history of dyslexia.

^a^ Presented as χ^2^.

### Psychometric and Environmental Characteristics

**Figure 2.**
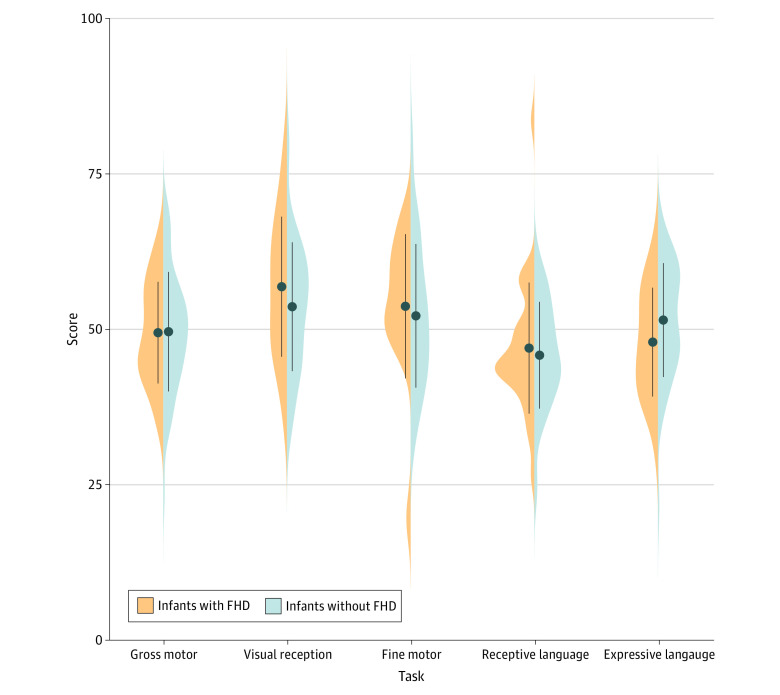
Behavioral Milestones of Infants With and Without Family History of Dyslexia (FHD) T-scores are presented for all 5 domains of the Mullen Scales of Early Learning assessment for infants with and without FHD (mean [SD] T-score, 50 [10]). No significant group differences were identified for any of the 5 domains.

Psychometric results collected from 29 infants with FHD and 48 infants without FHD revealed no significant group differences in any developmental area (Figure 2). The 2 groups were also comparable in socioeconomic and HLE characteristics (except for a higher frequency of rhyme and joke sharing among the families with FHD) (eTable 1 in the Supplement).

### Classification Results of the Functional Connectivity Patterns

**Figure 3.**
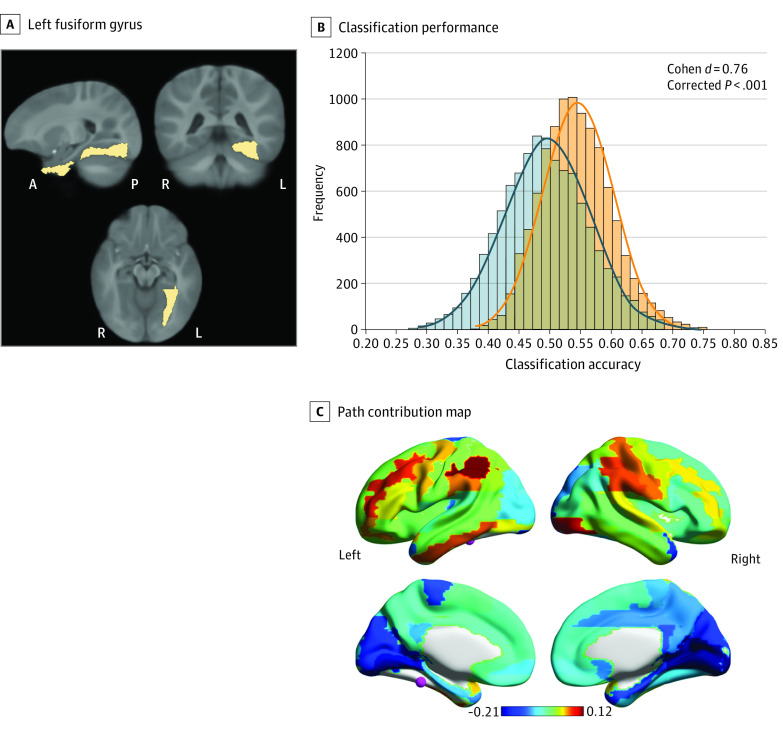
Distinct FC Patterns of Left Fusiform Gyrus Between Infants With vs Without Family History of Dyslexia (FHD) A, Slice views of the left fusiform gyrus. Brain orientations are marked at the bottom of each image. A indicates anterior; L, left; P, posterior; R, right. B, Distribution of classification accuracies based on the true group labels (orange) against the null distribution based on permutation results (blue). C, Contribution of each path to the familial risk classification performance by projecting the pattern value of each functional connectivity onto the corresponding cerebral region that left fusiform gyrus connects to. The seed region of left fusiform gyrus is highlighted as a node in pink color. Images were prepared using MRIcron and BrainNet software.^50^

Head movement during scanning was not different between infants with and without FHD in terms of the percentage of outlier images and the mean framewise displacement after outlier removal (Table). Among the 20 seed regions associated with language and reading development, classification analyses identified distinct FC patterns between infants with vs without FHD only in the left fusiform gyrus (LFFG; classification accuracy, 0.55 [99% CI, 0.047-0.052]; corrected *P* < .001; *d* = 0.76) (Figure 3; eTable 2 in the Supplement). The classification performance based on the FC patterns of LFFG also showed similar sensitivity (classification sensitivity, 0.54 [99% CI, 0.038-0.044]; corrected *P* < .001; *d* = 0.56) and specificity (classification specificity, 0.56 [99% CI, 0.054-0.060], corrected *P* < .001; *d* = 0.76) characteristics. Result replications with age as a covariate and using the 5-fold cross-validation approach are presented in the eAppendix in the Supplement). Moreover, the pattern value (contribution) of each path for this classification performance was computed and presented in Figure 3C. Connections with the highest positive pattern values linked LFFG to the bilateral inferior parietal, right inferior occipital, left middle frontal, and right supramarginal gyri, and those with greatest negative pattern values primarily connected LFFG to the medial occipital areas, such as bilateral lingual and calcarine gyri (eTable 3 in the Supplement). Finally, we additionally conducted 2-sample *t* tests between infants with and without FHD on the FC for connections between LFFG and each of the cerebral regions (ie, path-specific FC). While no path showed significant group differences, a significant positive correlation was identified between the pattern values and *t* values across all paths (*r*_75_ = 0.96; *P* < .001) (eFigure in the Supplement).

## Discussion

The findings of this cohort study suggest that functional connectivity alterations associated with familial risk of dyslexia were evident from as early as infancy. Using multivariate pattern analyses, distinct FC patterns of LFFG were identified in a cohort of 98 infants with and without FHD with well-balanced cognitive and environmental characteristics. Moreover, the LFFG connections to the regions constituting the frontal and parietal language and attention networks showed the highest positive pattern values, indicating significant contributions to the classification performance between infants with vs without FHD. To our knowledge, our study is the first to identify early FC alterations associated with FHD status in infancy long before a diagnosis would be possible based on early precursors or atypical reading performance.

Distinct FC patterns of LFFG between infants with vs without FHD suggest that some of the atypical network mechanisms associated with FHD may emerge early in development. The fusiform gyrus is a key component of the ventral occipitotemporal cortex (VOTC) supporting high-level visual recognition of categories in adults, such as faces (fusiform face area).^51^ The visual word form area (VWFA), specialized for visual word recognition, emerges within the LFFG at approximately the time of reading onset, indicating experience-dependent functional specialization.^52,53^ Yet, FC between the VOTC (housing the LFFG) and the left frontal and temporoparietal components of the language network is already present in prereaders^54^ from as early as birth.^55^ These early-emerging connections are postulated to provide an initial neural scaffold for establishing associations between word forms and their phonological and semantic content during literacy acquisition.^2,56^ Accordingly, the LFFG is also sensitive to grapheme-to-phoneme mapping essential for reading development.^57^ We have previously shown that the infant FC patterns of LFFG are longitudinally associated with phonological skills in children aged 6 years.^37^ This highlights the long-term cognitive significance of the LFFG and its FC on the trajectory of literacy development. Within this framework, the current observation of atypical FC of the LFFG in infants with FHD might indicate early alterations in the neural network mechanisms underlying the integration of visual word processing and other functions important for reading acquisition, such as oral language.

The path contribution results further support this postulation. The activation pattern of each path derived from the classification model reflects the direction and strength of the group effects embedded in each FC.^49^ In this study, despite no significant group differences in the path-specific FC, a significant correlation was shown between the pattern values and *t* values across all paths. This suggests that paths with larger group differences (in both directions) contributed greater to the classification. Along this line, we further identified that paths with the highest positive pattern values connected LFFG to regions that are part of the language and speech processing network (left middle frontal and right supramarginal gyri^58,59^) or constitute the parietal attention (bilateral inferior parietal^60^) and ventral visual (right inferior occipital) circuits in adults; however, their roles in the early development of these functions require further investigation. Moreover, these paths showed numerically higher FC in infants without FHD than in those with FHD, although the difference was not statistically significant. These results align with previously observed FC disruptions between VWFA and these networks in older children with dyslexia,^20^ supporting proposed associations between FHD and altered connectivity between the visual pathways and the language and attention circuits evident in infancy. Finally, paths connecting LFFG and the adjacent areas in bilateral lingual and calcarine gyri showed the greatest negative pattern values and displayed numerically higher FC in infants with FHD than in those without FHD. We hypothesize that such findings might indicate atypical topological organization of the medial occipital areas in infants with FHD, which should be re-evaluated using clustering-based techniques (ie, independent component analysis) in future investigations.

The atypical FC patterns of LFFG observed in infants with FHD further shed light on the developmental mechanisms underlying dyslexia-associated neural alterations of VWFA. VWFA hypoactivation is reported not only in adults with dyslexia^18,61^ but also among prereaders who subsequently developed dyslexia,^62^ suggesting neural deficits contributing to the onset of dyslexia. Nevertheless, it remains unclear what specific mechanism causes functional disruptions to print (eg, letters) in this area among prereaders. We have previously shown that the structural connectivity patterns of the VOTC in prereaders were associated with its functional tuning to words 3 years later after reading onset,^36^ supporting a critical role of interregional connectivity in shaping the functional specialization during reading development.^35,63^ Following this hypothesis, it can be postulated that the FHD-associated disruptions in the FC of LFFG might serve as a developmental mechanism underlying characteristic VWFA alterations associated with dyslexia (eAppendix in the Supplement). Notably, such FC alteration might also implicate the functional specialization for other visual categories, which could account for the atypical neural responses to face recognition reported in some individuals with dyslexia.^62,64^ Future studies are warranted to empirically examine the hypothesized link between infants’ functional organization of the VOTC and subsequent functional specialization of the VWFA and the association specificity to literacy development.

### Limitations

This study has some limitations. First, most infants were scanned within 7 to 10 minutes after falling asleep to ensure similar sleeping states during scanning. However, the sleeping state was not strictly controlled or monitored owing to technical challenges. It is therefore unclear whether our results are modulated by the infants’ sleeping states. However, it is unlikely that these sleeping states introduced a systematic group bias.^65^ Second, no HLE differences were observed between infants with vs without FHD, which is different from previously reported decreased HLE among older children with FHD compared with children without FHD.^66,67^ Most infant books have less text, simpler vocabulary, and less complex syntax than books for older children and, therefore, the FHD-associated differences in HLE may not be observable yet.^68^ Future studies with HLE measures applicable to a wider age range are needed to better understand environmental characteristics associated with family members with or without FHD. Third, the modest classification accuracies of this study limit the generalizability of its results. Although our findings are replicable using the 5-fold cross-validation approach with increased estimation reliabilities,^69^ objective reevaluation of these results is needed using independent data sets. With a larger sample size, future work could also conduct hyperparameter optimization based on a nested cross-validation approach to maximize classification performance.^70^ Additionally, it is important to note that while FHD-associated alterations have been repeatedly shown at the group level, children with FHD exhibit a full range of reading abilities, with only 40% to 60% developing dyslexia.^4^ These individual differences among children with FHD not only suggest a nuanced FHD pattern rather than 2 distinct groups, which possibly contributes to the limited predictive powers reported, but also indicate potential neural and environmental factors associated with facilitating reading acquisition across the developmental timeline.^43,71^ Building on the observed infant FC mechanisms associated with FHD, longitudinal follow-up studies will be critical in elucidating the neurodevelopmental trajectories of the characteristic neural alterations observed among infants with FHD. Such investigations should further consider more advanced statistical frameworks (eg, Bayesian approaches^72^) that enable the characterization of individual-level FC patterns underlying individual differences in behavioral measurements. It will be especially critical to investigate which children with FHD subsequently develop reading difficulties, as well as identify potentially protective mechanisms and resilience patterns in those who do not develop dyslexia. Addressing these critical questions will not only help to illuminate the neural etiology of dyslexia, but also has the potential to inform approaches to early diagnosis and intervention.

## Conclusions

This cohort study provides the first evidence, to our knowledge, of atypical functional networks in infants with a familial risk of dyslexia compared with those without, despite comparable developmental milestones and socioeconomic and environmental factors. These alterations were observed in the infant FC patterns of LFFG, an area that underlies long-term language and literacy development. Moreover, early disruptions were seen in connections linking LFFG to regions constituting the language and attention networks, which are hypothesized to play an important role in the functional specialization of the VWFA during reading acquisition. This work suggests that atypical functional topologies in individuals with a familial risk of dyslexia are observable as early as infancy. Longitudinal tracking will be critical for delineating how atypical functional networks in infancy may contribute to the manifestation of subsequent reading ability and for uncovering genetic and environmental factors underlying this developmental trajectory.

## Notes

Supplement. **eAppendix.** Additional Methods and Results **eTable 1.** Environmental Characteristics for Infants With and Without FHD **eTable 2.** Classification Results for All 20 Seed Regions Important for Long-term Language and Reading Development **eTable 3.** Path Patterns in the Familial Risk Classification of the Functional Connectivity Patterns of the Left Fusiform Gyrus **eFigure.** Scatter Plot for the Correlation Results Between the Pattern Values of Each Path Estimated From the Classification Model and *t* Values Derived From the 2-Sample Comparisons on the Path-Specific FC
eReferences.
