# Supplementary material for: Patterns of Neural Functional Connectivity in Infants at Familial Risk of Developmental Dyslexia
Source: JAMA Netw Open. 2022 Oct 27;5(10):e2236102. doi: 10.1001/jamanetworkopen.2022.36102 (PMC9614583; doi:10.1001/jamanetworkopen.2022.36102)
Supplement: Supplement. — eAppendix. Additional Methods and Results eTable 1. Environmental Characteristics for Infants With and Without FHD eTable 2. Classification Results for All 20 Seed Regions Important for Long-term Language and Reading Development eTable 3. Path Patterns in the Familial Risk Classification of the Functional Connectivity Patterns of the Left Fusiform Gyrus eFigure. Scatter Plot for the Correlation Results Between the Pattern Values of Each Path Estimated From the Classification Model and t Values Derived From the 2-Sample Comparisons on the Path-Specific FC eReferences. [file jamanetwopen-e2236102-s001.pdf]

Supplemental Online Content

Yu X, Ferradal S, Dunstan J, et al. Patterns of neural functional connectivity in infants at familial risk of developmental dyslexia. *JAMA Netw Open*. 2022;5(10):e2236102. doi:10.1001/jamanetworkopen.2022.36102

eAppendix. Additional Methods and Results
eTable 1. Environmental Characteristics for Infants With and Without FHD
eTable 2. Classification Results for All 20 Seed Regions Important for Long-term Language and Reading Development
eTable 3. Path Patterns in the Familial Risk Classification of the Functional Connectivity Patterns of the Left Fusiform Gyrus
eFigure. Scatter Plot for the Correlation Results Between the Pattern Values of Each Path Estimated From the Classification Model and t Values Derived From the 2-Sample Comparisons on the Path-Specific FC
eReferences.

This supplemental material has been provided by the authors to give readers additional information about their work.

eAppendix. Additional Methods and Results

Participant recruitment.

All infants were selected from an ongoing longitudinal study at Boston Children's Hospital (BCH; the study is now based at Harvard University) that aims to characterize neural trajectories underlying language and reading development from infancy to school-age among children with and without familial risk of dyslexia. Therefore, comprehensive background information required for assessing the familial risk of dyslexia was collected from each family upon participation. Specifically, parents were asked to specify which family member (mother, father and/or siblings) had a dyslexia diagnosis or reading difficulties, the date and location in case of formal diagnosis, and their follow-up treatment plans. Based on this information, infants were divided into FHD+ and FHD- groups.

A total of 118 participants have successfully completed resting-state functional and structural MRI sequences at infancy between 4 and 13 months of age. Among them, 20 subjects were excluded from further analyses due to poor image quality (n=5), atypical brain anatomy (n=2), and excessive head motion during imaging sessions (n=13, see details in the **Resting-state fMRI preprocessing** section), resulting in the final set of 98 subjects (35 FHD+, 63 FHD-) with usable resting-state fMRI data included in the current analyses.

Imaging acquisition.

Infant participants were scanned during natural sleep (N. Raschle et al., 2012) on a Siemens 3T Trio scanner with a 32-channel adult head coil. High-quality structural images were acquired using a motion-compensated multi echo MPRAGE sequence with the following parameters: slice number = 176, TR = 2270 ms, TE1,2,3,4 = [1.66, 3.48, 5.3, 7.12] ms, flip angle = 7◦, TI = 1450 ms, field of view= 220 mm^2, voxel size =1.1×1.1×1.0 mm^3. An 8-minute blood-oxygen level dependent (BOLD) weighted imaging was further collected with two acquisition sequences: for data acquired earlier, the parameters were TR = 3000 ms, TE = 30 ms, flip angle = 60°, voxel size =3 x 3 x 3 mm^3; whereas an additional simultaneous multi-slice (SMS) imaging technique with a short TR (950 ms) was applied to the later collected images (the other parameters remained the same). The ratio of the FHD+ and FHD- infants were equivalent across two acquisition sequences ($X^2 = 0.11$, $p = 0.74$).

Resting-state fMRI preprocessing.

Preprocessing was conducted following an infant-specific preprocessing pipeline implemented in the FSL toolbox (Smith et al., 2004, also see details in Yu et al., 2021). Structural images were first skull-stripped and segmented into gray matter, white matter (WM), and cerebral spinal fluid (CSF). FMRI images were corrected for slice timing and head movement, and then normalized to the UNC 1-year old infant template (Shi et al., 2011) via the high-resolution structural image of the same infant using affine transformations. Based on the head movement parameters obtained during motion correction, framewise displacement (FD) was computed (Power et al., 2012) using an in-house script (https://github.com/xiyu-bnu/infant_restingstate_prediction). Outlier volumes identified as FD > 0.3 mm were marked with one preceding and two subsequent frames to minimize the spreading effect due to temporal filtering or spin history. Thirteen infant participants with less than 5-min usable volumes after outlier removal were excluded from the subsequent analyses. All participants included in the final analyses had an average of 7.0% outlier images. Outlier images were then coded as a binary vector and submitted into linear regression with the six continuous motion regressors and mean CSF and WM signals estimated based on the subject-specific anatomical masks. After linear regression, images were temporally band-pass filtered (0.01-0.1Hz) and spatially smoothed (Gaussian filter, FWHM = 6mm). Finally, the fMRI time series with multi-slice acquisition were further temporally resampled to a TR = 3000ms in order to keep a consistent temporal resolution across the whole sample.

Replication analyses using age as a covariate.

Significant brain changes within the first year of life might impose a significant impact on the registration process of the acquired T1 images to the templates and on the subsequent fMRI image normalization. However, these potential challenges are comparable between the FHD+ and FHD- infants in the current study, given the non-significant differences in age at scan between the two groups (FHD+ = 8.9 ± 2.4 months, FHD- = 8.3 ± 2.3 months, $t_{96} = 1.1$, $p = 0.25$). Nevertheless, to ensure that the distinct functional connectivity patterns of LFFG between FHD+ and FHD- infants were independent of age, we further conducted replication analyses with age as a covariate. To this aim, during the leave-one-pair-out cross-validation analyses, the age effect was regressed out from each input feature (i.e., functional connectivity between LFFG and each cerebral region) of the training dataset using a linear regression model. The residuals of these models, representing the individual differences in the FC patterns independent of potential age influences, were then entered into the SVM analyses. Moreover, each estimated linear model was further applied to the corresponding feature of the testing dataset (i.e., the held-out pair) to obtain the age-independent FC patterns (i.e., model residuals) of the testing participants. The predicted FHD labels were then generated by submitting

these residual values to the trained SVM model, which were compared with the true labels of the testing data for classification performance estimation. Permutation tests were conducted following the same procedure with the exception that FHD labels were randomized across participants. Similar to the results reported in the main manuscript, a higher-than-chance-level classification performance was revealed for the FC patterns of the left fusiform gyrus (accuracy = 0.54, $p_{corrected} < 0.001$, 99% CI of the classification performance = [0.038, 0.042], Cohen's $d = 0.61$; sensitivity = 0.53, $p_{corrected} < 0.001$, 99% CI of the classification performance = [0.029, 0.034], Cohen's $d = 0.42$; specificity = 0.55, $p_{corrected} < 0.001$, 99% CI of the classification performance = [0.046, 0.051], Cohen's $d = 0.64$), demonstrating that the current observation of atypical FC patterns of LFFG in FHD+ compared to the control infants is independent of age.

Replication analyses using 5-fold cross-validation approach.

To evaluate the stability of the results obtained from the main analyses based on the leave-one-pair-out cross-validation approach, the identified seed region (i.e., LFFG) with distinct FC patterns between FHD+ and FHD- infants was re-evaluated using the 5-fold cross-validation approach. Similar to the main analyses, the bootstrapping approach was first applied to randomly select 35 infants from the FHD- pool, which were combined with the 35 FHD+ infants for the SVM analyses, and this process was run 10,000 times to reduce sampling bias. In each sampling (iteration), all data were equally divided into five folds with 7 FHD+ and 7 FHD- infants each. Data of four folds (i.e., 28 FHD+ and 28 FHD- infants) were used to estimate the SVM model (i.e., the training dataset), while the data of the remaining fold (i.e., 7 FHD+ and 7 FHD- infants) were submitted to the trained SVM model to produce the predicted labels (i.e., the testing dataset). The same procedure repeated five times so that every fold (and every infant) was used as the testing data once, generating the corresponding predicted labels for the evaluation of the classification accuracy. A true distribution was generated based on 10,000 iterations which was compared against the null distribution derived from permutation tests similarly run with randomized labels. The 5-fold cross-validation approach demonstrated a similarly higher-than-chance-level classification performance for the FC patterns of LFFG (accuracy = 0.55, $p_{corrected} < 0.001$, 99% CI of the classification performance = [0.045, 0.049], Cohen's $d = 0.75$; sensitivity = 0.54, $p_{corrected} < 0.001$, 99% CI of the classification performance = [0.034, 0.040], Cohen's $d = 0.45$; specificity = 0.56, $p_{corrected} < 0.001$, 99% CI of the classification performance = [0.055, 0.060], Cohen's $d = 0.72$), replicating the distinct FC patterns of LFFG between FHD+ and FHD- infants reported in the main manuscript (using the leave-one-pair-out cross-validation approach). Finally, the pattern value (contribution) of each path derived from the 5-fold cross-validation approach also showed high consistence with that computed in the original analyses ($r = 0.99$), demonstrating reproducibility of the path/feature pattern results.

Evaluation of the associations between the FC pattern alterations and familial risk of dyslexia in the anterior and posterior segments of LFFG.

Recent studies have suggested a posterior-anterior segregation in the visual word form area (VWFA) and its adjacent area for written language recognition in adults (Caffarra et al., 2021; Lerma-Usabiaga et al., 2018). Specifically, while the posterior segment is responsible for extracting the defining visual features of words, the anterior segment, including the classic VWFA peak, is primarily involved in processing the linguistic properties of the written stimuli, presumably through its connectivity with the language network. Therefore, to further evaluate whether the identified FC alterations of the entire LFFG (housing the VWFA) might be particularly predominant in either segment, the anterior and posterior segments of the infant LFFG were created and classification analyses were reperformed with each of the two segments as the seed region. Note that based on previous literature (Caffarra et al., 2021; Lerma-Usabiaga et al., 2018), these two functionally distinctive segments should be separated at y=-60 in the adult space. To achieve anatomically-equivalent segments in the infant space, we first extracted the left fusiform gyrus ROI from the adult AAL atlas (Tzourio-Mazoyer et al., 2002) and divided it into the anterior and posterior segments at y=-60. These two segments were then warped back from the MNI templates to the one-year-old infant templates using Advanced Normalization Tools (ANTs, Avants et al., 2009), and were further overlapped with the LFFG ROI extracted from the one-year-old Infant Brain Atlases (Shi et al., 2011) to generate the final anterior and posterior LFFG segments in the one-year-old infant brain space. Whole brain FC patterns associated with each segment were calculated for every participant and submitted into the classification analyses with the same procedure described in the main manuscript. Such analyses revealed significant higher-than-chance classification performance with small effect sizes for both segments (anterior LFFG: accuracy = 0.52, $p_{corrected} < 0.001$, 99% CI of the classification performance = [0.021, 0.026], Cohen's $d = 0.36$; sensitivity = 0.54, $p_{corrected} < 0.001$, 99% CI of the classification performance = [0.036, 0.042], Cohen's $d = 0.51$; specificity = 0.51, $p_{corrected} < 0.001$, 99% CI of the classification performance = [0, 0.012], Cohen's $d = 0.11$; posterior LFFG: accuracy = 0.52, $p_{corrected} < 0.001$, 99% CI of the classification performance = [0.016, 0.021], Cohen's $d = 0.29$; sensitivity = 0.52, $p_{corrected} < 0.001$, 99% CI of the classification performance = [0.019, 0.025],

© Yu X et al. *JAMA Network Open*.

Cohen's *d* = 0.30; specificity = 0.51, $p_{corrected} < 0.001$, 99% CI of the classification performance = [0.013, 0.018], Cohen's *d* = 0.21). Importantly, the effect size of the differences in the classification accuracies of the two segments were negligible ($p < 0.001$, Cohen's $d = 0.09$), while they were both lower than those derived from the whole LFFG region by small (anterior LFFG: $p < 0.001$, Cohen's $d = 0.42$) or median (posterior LFFG: $p < 0.001$, Cohen's $d = 0.55$) effect size. These results thus speak against a strong differentiation of the anterior and posterior segments of LFFG in their infant FC associations with familial risk of dyslexia. Previous studies have shown that functional specialization for visual word recognition emerges within the LFFG after reading onset in both children and adults, indicating experience-dependent functional specialization (Brem et al., 2010; Dehaene et al., 2010). Moreover, widespread hypoactivation in the VWFA vicinity, including both anterior and posterior segments, has been observed in adults with dyslexia (e.g., Martin et al., 2016) as well as children at familial risk of dyslexia (Dębska et al., 2016). Our observation aligns with these findings, and further suggests the early onset of atypical FC patterns of the whole LFFG that are associated with familial risk of dyslexia, which might serve as the developmental mechanisms underlying dyslexia-associated neural alterations of the VWFA and its vicinity. Future studies are needed to further elucidate whether deficits in the anterior and posterior segments of the VWFA vicinity observed in dyslexia might be associated with different neural and cognitive characteristics, and possibly exhibit distinctive emerging mechanisms over development.

© Yu X et al. *JAMA Network Open*.

eTable 1. Environmental Characteristics for Infants With and Without FHD

Questions for environmental characterization		Whole sample		
		FHD- (%)	FHD+ (%)	Group comparison (Mann–Whitney U test)
Home literacy environment				
1. Total number of parent/adult books in the home	0-10	6.3	5.7	*p* = 0.53
	11-50	15.9	11.4	
	51-100	19.0	22.9	
	101-200	20.6	22.9	
	201-300	17.5	5.7	
	>300	17.5	28.6	
	N/A	3.2	2.9	
2. Total number of children's books in the home	0-10	0.0	2.9	*p* = 0.44
	11-50	30.2	25.7	
	51-100	22.2	34.3	
	101-200	22.2	14.3	
	201-300	7.9	11.4	
	>300	14.3	8.6	
	N/A	3.2	2.9	
3. Age (in months) of child when first read to	Prenatal	19.4	25.0	*p* = 0.59
	Less than one	35.5	18.8	
	1-2	25.8	25.0	
	3-5	9.7	12.5	
	6-9	0.0	6.3	
	10 or more	3.2	0.0	
	N/A	6.5	12.5	
4. Amount of time at home that someone reads to child (hours/week)	Less than one	7.9	5.7	*p* = 0.39
	1	19.0	22.9	
	2	19.0	14.3	
	3	19.0	28.6	
	4-5	19.0	5.7	
	6 or more	7.9	14.3	
	N/A	7.9	8.6	
5. How often do family members teach the child to count? (times/week)	Never	27.0	28.6	*p* = 0.48
	1-2	14.3	25.7	
	3-4	14.3	20.0	
	5-6	4.8	0.0	
	Daily	23.8	20.0	
	N/A	15.9	5.7	
6. How often do family members teach the child the alphabet? (times/week)	Never	23.8	25.7	*p* = 0.28
	1-2	20.6	37.1	
	3-4	14.3	8.6	
	5-6	6.3	0.0	
	Daily	19.0	17.1	
	N/A	15.9	11.4	

© Yu X et al. *JAMA Network Open*.

7. How often do family members read newspapers, books, or magazines? (times/week)	Never	6.3	8.6	*p* = 0.082
	1-2	9.5	11.4	
	3-4	14.3	2.9	
	5-6	6.3	22.9	
	Daily	58.7	51.4	
	N/A	4.8	2.9	
8. How often do family members write messages, notes, or lists? (times/week)	Never	3.2	5.7	*p* = 0.11
	1-2	3.2	5.7	
	3-4	4.8	20.0	
	5-6	12.7	5.7	
	Daily	73.0	60.0	
	N/A	3.2	2.9	
9. How often do family members write letters, cards, diaries, stories, or poems? (times/week)	Never	9.5	14.3	*p* = 0.47
	1-2	63.5	48.6	
	3-4	11.1	11.4	
	5-6	1.6	0.0	
	Daily	7.9	17.1	
	N/A	6.3	8.6	
10. How often do family members share rhymes or jokes orally with the child? (times/week)	Never	7.9	2.9	*p* = 0.015
	1-2	15.9	2.9	
	3-4	4.8	25.7	
	5-6	3.2	2.9	
	Daily	61.9	62.9	
	N/A	6.3	2.9	
Socio-economic status				
Maternal highest educational degree	8th Grade or Less	0.0	0.0	*p* = 0.35
	HS/GED	4.8	11.4	
	Associate Degree	3.2	5.7	
	Bachelor’s Degree	31.7	14.3	
	Master’s Degree	41.3	42.9	
	Doctorate or equivalent	19.0	17.1	
	N/A	0.0	8.6	
Paternal highest educational degree	8th Grade or Less	0.0	2.9	*p* = 0.32
	HS/GED	11.1	17.1	
	Associate Degree	4.8	8.6	
	Bachelor’s Degree	31.7	14.3	
	Master’s Degree	25.4	20.0	
	Doctorate or equivalent	25.4	22.9	
	N/A	1.6	14.3	

For items with multiple choices, response frequency for each option (in percentage) was listed. Given the ordinal nature of parental response, group effects were examined using the Wilcoxon–Mann–Whitney two-sample rank-sum tests after excluding the “N/A” responses.

FHD-: infants without familial history of dyslexia; FHD+: infants with familial history of dyslexia

© Yu X et al. *JAMA Network Open*.

eTable 2. Classification Results for All 20 Seed Regions Important for Long-term Language and Reading Development

	Accuracy			Sensitivity			Specificity		
	**Mean**	**99% CI (CL performance)**	**Cohen *d***	**Mean**	**99% CI (CL performance)**	**Cohen *d***	**Mean**	**99% CI (CL performance)**	**Cohen *d***
Left Hemisphere									
Pars Orbitalis	0.48	[-0.020, -0.015]		0.47	[-0.035, -0.029]		0.50	[-0.006, -0.001]	
Pars Triangularis	0.46	[-0.042, -0.038]		0.48	[-0.025, -0.020]		0.44	[-0.060, -0.055]	
Pars Opercularis	0.42	[-0.078, -0.074]		0.44	[-0.062, -0.057]		0.41	[-0.095, -0.089]	
Precentral Gyrus	0.46	[-0.041, -0.036]		0.48	[-0.024, -0.019]		0.45	[-0.057, -0.052]	
Heschl’s Gyrus	0.50	[-0.004, 0.001]		0.51	[0.003, 0.008]		0.49	[-0.011, -0.006]	
Inferior Parietal Gyrus	0.46	[-0.047, -0.042]		0.47	[-0.035, -0.030]		0.44	[-0.060, -0.054]	
Supramarginal Gyrus	0.47	[-0.029, -0.024]		0.48	[-0.022, -0.017]		0.47	[-0.036, -0.031]	
Angular Gyrus	0.49	[-0.014, -0.009]		0.49	[-0.010, -0.005]		0.48	[-0.017, -0.012]	
Fusiform Gyrus	0.55*	[0.047, 0.052]	0.76	0.54	[0.038, 0.044]	0.56	0.56	[0.054, 0.060]	0.76
Inferior Temporal Gyrus	0.53*	[0.029, 0.034]	0.49	0.51	[0.005, 0.010]	0.10	0.56	[0.053, 0.058]	0.76
Right Hemisphere									
Pars Orbitalis	0.49	[-0.015, -0.010]		0.49	[-0.015, -0.010]		0.49	[-0.015, -0.010]	
Pars Triangularis	0.48	[-0.025, -0.021]		0.49	[-0.016, -0.010]		0.47	[-0.035, -0.030]	
Pars Opercularis	0.50	[-0.0051, -0.0005]		0.49	[-0.009, -0.003]		0.50	[-0.002, 0.003]	
Precentral Gyrus	0.52*	[0.013, 0.018]	0.24	0.49	[-0.017, -0.011]		0.55	[0.043, 0.048]	0.61
Heschl’s Gyrus	0.50	[-0.003, 0.002]		0.47	[-0.030, -0.024]		0.53	[0.023, 0.029]	
Inferior Parietal Gyrus	0.48	[-0.027, -0.023]		0.48	[-0.028, -0.022]		0.47	[-0.028, -0.023]	
Supramarginal Gyrus	0.51	[0.005, 0.009]		0.53	[0.022, 0.028]		0.49	[-0.014, -0.008]	
Angular Gyrus	0.45	[-0.056, -0.052]		0.46	[-0.043, -0.038]		0.43	[-0.070, -0.065]	
Fusiform Gyrus	0.46	[-0.038, -0.033]		0.48	[-0.025, -0.020]		0.45	[-0.051, -0.045]	

© Yu X et al. *JAMA Network Open*.

Inferior Temporal Gyrus	0.49	[-0.010, -0.005]	0.51	[0.009, 0.014]	0.47	[-0.029, -0.024]

Note. Among the 20 seed regions previously identified to be associated with language and reading development, only the left fusiform gyrus (highlighted in red) showed distinct functional connectivity patterns between FHD+ and FHD- infants, demonstrated by the above-chance-level classification accuracy, sensitivity and specificity with a median or large effect size (Cohen's d > 0.5), as well as positive 99% confidence intervals (CI) of the classification performance.

* Classification performance based on the real group information significantly (after correcting for multiple comparisons) outperformed the null results derived from the randomized labels ($p_{corrected} < 0.05$)

© Yu X et al. *JAMA Network Open*.

eTable 3. Path Patterns in the Familial Risk Classification of the Functional Connectivity Patterns of the Left Fusiform Gyrus

Cerebral cortices	Pattern values (mean ± standard deviation)
Left inferior parietal lobule	0.112 ± 0.050
Right inferior occipital gyrus	0.089 ± 0.041
Left middle frontal gyrus	0.069 ± 0.052
Right inferior parietal lobule	0.066 ± 0.046
Right supramarginal gyrus	0.063 ± 0.041
Left inferior temporal gyrus	0.060 ± 0.039
Right postcentral gyrus	0.055 ± 0.040
Left supramarginal gyrus	0.052 ± 0.044
Left orbitofrontal cortex (middle)	0.049 ± 0.048
Left precentral gyrus	0.042 ± 0.042
Left temporal pole (superior)	0.039 ± 0.054
Right middle frontal gyrus	0.034 ± 0.051
Right temporal pole (superior)	0.033 ± 0.052
Right superior temporal gyrus	0.031 ± 0.051
Left inferior occipital gyrus	0.029 ± 0.041
Left inferior frontal gyrus (triangular)	0.028 ± 0.053
Right orbitofrontal cortex (middle)	0.024 ± 0.050
Right precentral gyrus	0.020 ± 0.045
Right inferior temporal gyrus	0.010 ± 0.040
Right superior parietal gyrus	0.010 ± 0.051
Left angular gyrus	0.009 ± 0.045
Left superior parietal gyrus	0.004 ± 0.062
Left inferior frontal gyrus (opercular)	0.004 ± 0.047
Left superior frontal gyrus (dorsal)	0.001 ± 0.047
Left postcentral gyrus	0.000 ± 0.043
Left orbitofrontal cortex (inferior)	-0.001 ± 0.049
Left middle temporal gyrus	-0.003 ± 0.051
Left orbitofrontal cortex (superior)	-0.004 ± 0.043
Right Heschl gyrus	-0.006 ± 0.0481
Left insula	-0.007 ± 0.049
Right rolandic operculum	-0.008 ± 0.042
Right orbitofrontal cortex (superior)	-0.011 ± 0.048
Right middle temporal gyrus	-0.011 ± 0.045
Right inferior frontal gyrus (triangular)	-0.028 ± 0.050
Left supplementary motor area	-0.028 ± 0.042
Right superior frontal gyrus (dorsal)	-0.032 ± 0.044
Left olfactory	-0.033 ± 0.051
Right orbitofrontal cortex (inferior)	-0.033 ± 0.048
Right insula	-0.034 ± 0.048
Left middle cingulate gyrus	-0.036 ± 0.037
Left rolandic operculum	-0.036 ± 0.043
Right middle occipital gyrus	-0.037 ± 0.047
Left superior frontal gyrus (medial)	-0.038 ± 0.040
Left superior temporal gyrus	-0.038 ± 0.049
Left anterior cingulate gyrus	-0.040 ± 0.043
Left precuneus	-0.041 ± 0.057
Right supplementary motor area	-0.044 ± 0.045
Right anterior cingulate gyrus	-0.045 ± 0.045
Right inferior frontal gyrus (opercular)	-0.045 ± 0.043
Right superior frontal gyrus (medial)	-0.050 ± 0.042

© Yu X et al. *JAMA Network Open.*

Left rectus gyrus	-0.051 ± 0.041
Right rectus gyrus	-0.054 ± 0.044
Right angular gyrus	-0.055 ± 0.041
Right orbitofrontal cortex (medial)	-0.060 ± 0.040
Left superior occipital gyrus	-0.062 ± 0.062
Right fusiform gyrus	-0.069 ± 0.038
Left orbitofrontal cortex (medial)	-0.070 ± 0.038
Left middle occipital gyrus	-0.070 ± 0.052
Left posterior cingulate gyrus	-0.081 ± 0.0423
Right paracentral lobule	-0.081 ± 0.049
Left paraHippocampal gyrus	-0.081 ± 0.046
Right superior occipital gyrus	-0.087 ± 0.063
Right precuneus	-0.089 ± 0.057
Right middle cingulate gyrus	-0.092 ± 0.042
Right olfactory	-0.093 ± 0.050
Left temporal pole (middle)	-0.096 ± 0.048
Left Heschl gyrus	-0.099 ± 0.047
Right posterior cingulate gyrus	-0.121 ± 0.042
Right paraHippocampal gyrus	-0.122 ± 0.046
Left paracentral lobule	-0.124 ± 0.053
Left calcarine cortex	-0.143 ± 0.060
Right cuneus	-0.145 ± 0.0622
Right temporal pole (middle)	-0.145 ± 0.049
Left cuneus	-0.158 ± 0.059
Left lingual gyrus	-0.192 ± 0.043
Right calcarine cortex	-0.207 ± 0.052
Right lingual gyrus	-0.210 ± 0.045

© Yu X et al. *JAMA Network Open*.

eFigure. Scatter Plot for the Correlation Results Between the Pattern Values of Each Path Estimated From the Classification Model and t Values Derived From the 2-Sample Comparisons on the Path-Specific FC

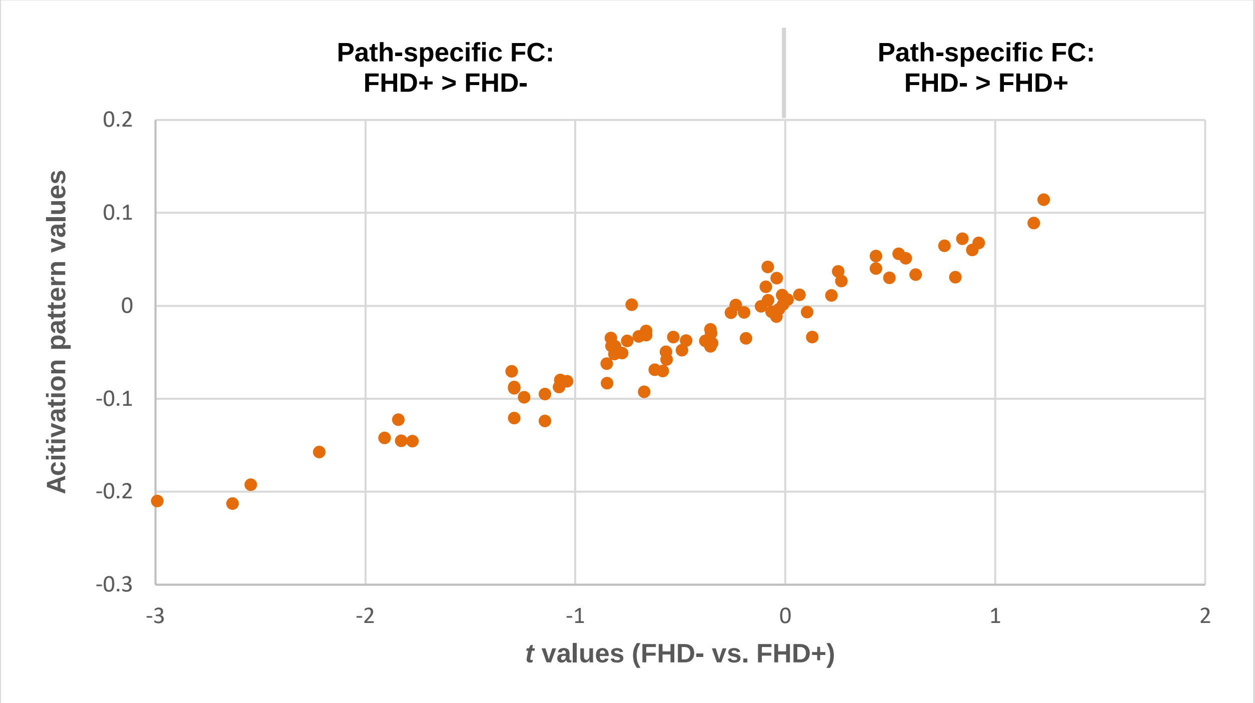

Note. None of the FC connections between left fusiform gyrus and each cerebral region (i.e., path-specific FC) showed significant group differences between infants with and without FHD ($p_{corrected}$ > 0.25). The positive *t* values indicate numerically higher FC for infants with than without FHD, and vice versa for the negative *t* values.
FHD-: infants without familial history of dyslexia; FHD+: infants with familial history of dyslexia

© Yu X et al. *JAMA Network Open*.

eReferences.

Avants, B. B., Tustison, N., & Song, G. (2009). Advanced normalization tools (ANTS). *Insight j*, 2(365), 1-35.

Brem, S., Bach, S., Kucian, K., Kujala, J. V., Guttorm, T. K., Martin, E., ... & Richardson, U. (2010). Brain sensitivity to print emerges when children learn letter–speech sound correspondences. *Proceedings of the National Academy of Sciences*, 107(17), 7939-7944.

Caffarra, S., Karipidis, I. I., Yablonski, M., & Yeatman, J. D. (2021). Anatomy and physiology of word-selective visual cortex: from visual features to lexical processing. *Brain Structure and Function*, 226(9), 3051-3065.

Dębska, A., Łuniewska, M., Chyl, K., Banaszkiewicz, A., Żelechowska, A., Wypych, M., ... & Jednoróg, K. (2016). Neural basis of phonological awareness in beginning readers with familial risk of dyslexia—Results from shallow orthography. *NeuroImage*, 132, 406-416.

Dehaene, S., Pegado, F., Braga, L. W., Ventura, P., Filho, G. N., Jobert, A., ... & Cohen, L. (2010). How learning to read changes the cortical networks for vision and language. *Science*, 330(6009), 1359-1364.

Lerma-Usabiaga, G., Carreiras, M., & Paz-Alonso, P. M. (2018). Converging evidence for functional and structural segregation within the left ventral occipitotemporal cortex in reading. *Proceedings of the National Academy of Sciences*, 115(42), E9981-E9990.

Martin, A., Kronbichler, M., & Richlan, F. (2016). Dyslexic brain activation abnormalities in deep and shallow orthographies: A meta−analysis of 28 functional neuroimaging studies. *Human brain mapping*, 37(7), 2676-2699.

Power, J. D., Barnes, K. A., Snyder, A. Z., Schlaggar, B. L., & Petersen, S. E. (2012). Spurious but systematic correlations in functional connectivity MRI networks arise from subject motion. *Neuroimage*, *59*(3), 2142-2154.

Shi, F., Yap, P. T., Wu, G., Jia, H., Gilmore, J. H., Lin, W., & Shen, D. (2011). Infant brain atlases from neonates to 1-and 2-year-olds. *PloS one*, 6(4), e18746.

Smith, S. M., Jenkinson, M., Woolrich, M. W., Beckmann, C. F., Behrens, T. E., Johansen-Berg, H., ... & Matthews, P. M. (2004). Advances in functional and structural MR image analysis and implementation as FSL. *Neuroimage*, *23,* S208-S219.

Tzourio-Mazoyer, N., Landeau, B., Papathanassiou, D., Crivello, F., Etard, O., Delcroix, N., ... & Joliot, M. (2002). Automated anatomical labeling of activations in SPM using a macroscopic anatomical parcellation of the MNI MRI single-subject brain. *Neuroimage*, 15(1), 273-289.

Yu, X., Ferradal, S. L., Sliva, D. D., Dunstan, J., Carruthers, C., Sanfilippo, J., ... & Gaab, N. (2021). Functional connectivity in infancy and toddlerhood predicts long-term language and preliteracy outcomes. *Cerebral Cortex*, 2021,1-12.

© Yu X et al. *JAMA Network Open*.
